# Computer-based quality of life questionnaires may contribute to doctor–patient interactions in oncology

**DOI:** 10.1038/sj.bjc.6600001

**Published:** 2002-01-07

**Authors:** G Velikova, J M Brown, A B Smith, P J Selby

**Affiliations:** Imperial Cancer Research Fund Cancer Medicine Research Unit, St James's University Hospital, Beckett Street, Leeds LS9 7TF, UK; Northern and Yorkshire Clinical Trials and Research Unit, 17 Springfield Mount, Leeds LS2 9NG, UK

**Keywords:** quality of life, individual, cancer, computer, communication

## Abstract

It is well recognized that oncologists should consider patients' quality of life and functioning when planning and delivering anticancer treatment, but a comprehensive assessment of how a patient feels requires a thorough inquiry. A standardized measurement of patients' quality of life may support clinicians in identifying important problems for discussion during the limited time of the medical consultations. The aim of this study was to assess the feasibility of computer-administered individual quality of life measurements in oncology clinics with immediate feedback of results to clinicians and to examine the impact of the information on consultations. The study employed a prospective non-randomized design with pre-test post-test within subjects comparisons and involved three medical oncologists and 28 cancer patients receiving chemotherapy. The intervention consisted of completion of quality of life questionnaires before the consultations and informing clinicians of the results. The main outcome measures were patients' perceptions of the content of baseline and intervention consultations and satisfaction with communication. A qualitative analysis of clinicians' interviews was performed. When clinicians had the quality of life results they enquired more often about daily activities (Z=−2.71, *P*=0.007), emotional problems (Z=−2.11, *P*=0.035) and work related issues (Z=−1.89, *P*=0.058). There was an increase in the number of issues discussed during the intervention consultation (Z=−1.89, *P*=0.059). Patients were highly satisfied with both consultations. The computer measurement was well accepted by patients who felt that the questionnaires were a useful tool to tell the doctors about their problems. The clinicians perceived that the quality of life data broadened the range of the clinical inquiry and helped them identify issues for discussion. Having symptoms and functional problems expressed quantitatively on a scale was useful for detection of change over time.

*British Journal of Cancer* (2002) **86**, 51–59. DOI: 10.1038/sj/bjc/6600001 www.bjcancer.com

© 2002 The Cancer Research Campaign

## 

Transfer of information between healthcare professionals and their patients, in both directions will always remain a critically important element in diagnosis, management and patient support. For cancer patients the issues surrounding their quality of life (QL) are recognized to be central to good patient care. Comprehensive assessment of how a patient feels and functions requires a thorough inquiry. The traditional medical history and physical examination are often insufficient for assessing the full range of health-related problems in cancer patients and in chronically ill patients in general (Calkins *et al*, 1991; Passik *et al*, 1998). A standardized measurement of patients' symptoms and functioning offers an alternative structured way of collecting subjective information.

Advances in health services research over the past decades have provided tools to measure the effects of illness on the daily lives of patients, in a standardized, reliable and valid way (Fitzpatrick *et al*, 1992; Ware, 1995). Multidimensional, self-reported questionnaires (usually called quality of life or health status questionnaires) are currently widely used in clinical research (Fayers *et al*, 1997). Several studies have investigated the value of paper-based questionnaires in clinical practice. These looked at patients with chronic diseases causing significant functional impairment and suggested that the health status reports provided accurate information, facilitated doctor-patient communication, but in general did not have a detectable impact on patients' functional status (Kazis *et al*, 1990; Calkins *et al*, 1994; Wagner *et al*, 1997). However, when resource and management suggestions were incorporated into the physicians' feedback, there was an improvement in emotional well-being and social functioning of the patients (Rubenstein *et al*, 1995). Two published studies in oncology addressed the impact of individual QL assessments on physicians' behaviour and doctor-patient communication (Detmar and Aaronson, 1998; Taenzer *et al*, 2000). The QL results appeared to stimulate physicians to initiate discussions on specific aspects of patients' health and well-being.

The implementation of standardized QL measurement in clinical practice is proving to be difficult due to practical, methodological and conceptual barriers (Deyo and Patrick, 1989; McHorney and Tarlov, 1995). The logistical problem of gathering real-time questionnaire data for immediate use in medical practice can be overcome by computer-based administration, scoring and presentation of QL results (Sigle and Porzsolt, 1996; Taenzer *et al*, 1997; Velikova *et al*, 1999). However, most clinicians are not trained in the evaluation of questionnaire data and may be uncertain how to interpret and respond to the results. How to present to clinicians individual patient's QL data in a way that helps them to interpret and use it efficiently, remains an important area of research. There is a need to study the steps that would follow the feedback of QL data to clinicians: how clinicians incorporate the information within the structure of the medical interview, does the additional information change their behaviour, do they find it clinically useful, or do they find any unfavourable effects. The evaluation of any new clinical intervention should also include an assessment of patients' attitude and perceptions of the impact on their care.

This project was therefore undertaken to assess the feasibility of using computer-administered individual QL measurement in oncology clinics with immediate feedback of results to clinicians and to examine the impact of the QL information on the content of the medical consultations and on patient satisfaction with communication. We will also describe patients' and physicians' acceptance and attitude to the process.

## METHODS

### Study design

This was a prospective non-randomized study using pre-test post-test within subjects comparisons. Cancer outpatients completed two standard questionnaires on a touch-screen computer on two occasions (baseline and intervention visits). Both times they were reviewed by the same physician. Graphic and numeric summaries of the results were given to the clinician only at the second intervention visit. After the visits patients were asked about the content of the consultations, their satisfaction with the visits and their attitude to the use of QL data. Semi-structured interviews were conducted with the clinicians after each intervention consultation and at the end of the study.

### Sample

Consecutive cancer patients receiving chemotherapy or biological therapy at the Medical Oncology Outpatient Clinics at St James's University Hospital, Leeds between October 1998 and March 1999 were considered for participation. Patients were eligible for the study if they were able to read and understand English, were willing to give informed consent and were expected to attend the clinic at least once after the baseline visit. Three clinicians (two consultants and one specialist registrar) were selected at random from all 10 medical oncologists at St James's Hospital. We aimed at a convenience sample of 10 patients per doctor which would allow us to detect a moderate effect size (0.5) (Cohen, 1988) of the intervention on patient satisfaction with 80% power and 5% significance level.

The project was approved by the Local Ethical Committee at St James's Hospital, Leeds. Written informed consent was obtained from all participating patients and clinicians.

### Intervention questionnaires

The European Organisation for Research and Treatment of Cancer – Core Quality of Life Questionnaire version 3.0 (EORTC QLQ-C30) and the Hospital Anxiety and Depression Scale (HADS) were used. The EORTC QLQ-C30 is a 30 item cancer-specific questionnaire including functional scales (physical, emotional, cognitive, social and role), a global health/QL scale, symptom scales (fatigue, pain, nausea/vomiting), and six single items assessing symptoms and financial impact of disease (Aaronson *et al*, 1993). The raw scores for the scales and items were linearly transformed to give standard scores in the range 0–100. Higher scores on the functioning and global health scales indicate better functioning, higher scores on the symptom scales represent a higher level of symptoms.

HADS is a 14-item instrument with two separate subscales for anxiety and depression. Scores range from 0–21 on each scale with higher scores indicating more distress. Scores above 11 suggest probable cases of anxiety or depressive illness and scores between 8 and 10 – borderline cases (Zigmond and Snaith, 1983).

**Figure 1:**
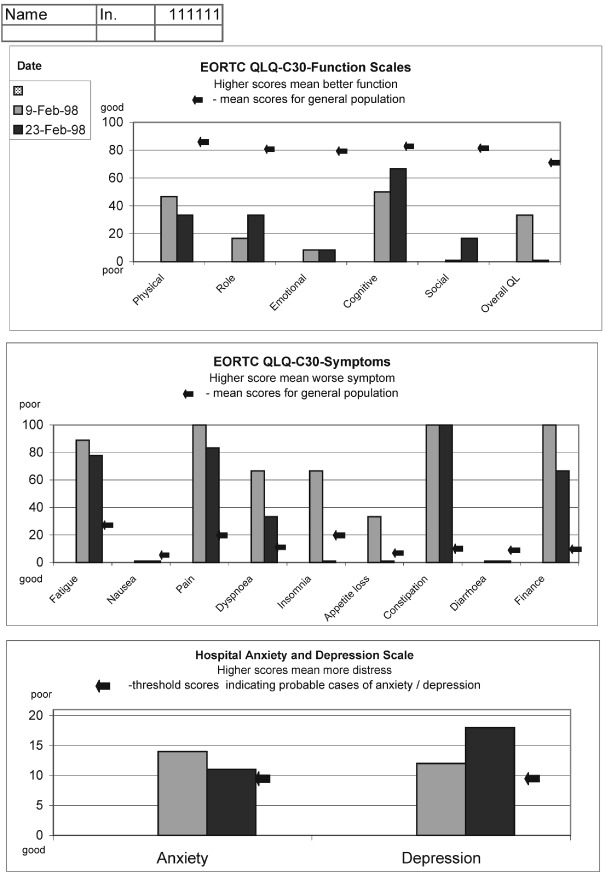
Example of the graphic print-out of the quality of life results.

Both questionnaires were administered in electronic format using computers with touch-screen monitors (the software was developed by AB Smith and can be obtained by writing to the author). The responses were scored immediately and the results were printed in two different formats – numerical (only present results) and graphical (incorporating present and previous results) (Figure 1

). The aim of having two different presentations of QL results was to assess clinicians' preferences.

#### Training clinicians in interpretation of QL results

A 1-h training session was conducted before the study was commenced. The training session focused on the content of EORTC QLQ-C30 and HADS, the interpretation of the scores and the general population reference data (Hjermstad *et al*, 1998; Fayers *et al*, 1998). Examples of individual patients profiles were discussed in conjunction with their clinical data.

### Outcome measures

#### Patients

Patient perceptions of the content of the consultation were assessed using a study specific checklist. It included seven possible discussion topics: (1) overall condition: (2) usual daily activities; (3) limitations in doing work or leisure activities; (4) how they feel emotionally; (5) symptoms of illness; (6) side effects of treatment; (7) impact of illness on relationships with family and friends. These topics were derived from the content of EORTC QLQ-C30. Patients were asked to indicate whether or not the doctor discussed any of these topics during the encounter.

Patient satisfaction with the clinic visit was assessed with a 17-item “Cancer Research Campaign Patient Satisfaction with Communication Questionnaire” (LJ Fallowfield, personal communication). This instrument was initially based on the 51 item Patient Satisfaction Questionnaire developed by Ware *et al* (quoted in Wilkin *et al*, 1992). It was extensively adapted and refined for use in UK in two pilot studies involving 289 and 154 oncology patients. The resulting 17-item questionnaire was psychometrically tested on a further sample of 629 cancer patients. The instrument includes direct statements of opinion about the consultation. Eight items are positively worded and nine negatively worded. Each item is scored from 1 (strongly agree) to 5 (strongly disagree). A high score indicates a high level of satisfaction. The range of scores is from 17 to 85. Principal components factor analysis revealed three dimensions: (1) satisfaction with rapport (six items – the doctor answered all questions, seemed to know what she/he was doing, handled the consultation well, did her/his best to keep me from worrying, seemed sympathetic, told me what I wanted to know); (2) dissatisfaction with doctor's manner (six items – the doctor could be irritated, could have been more respectful, too businesslike and impersonal, lacked experience with my medical problems, made me feel awkward, more attention to my privacy); (3) understanding (four items – the doctor used medical terms, the doctor told me all there was to know, satisfied with the medical care today, unclear about some things the doctor told me).

At the end of the study patient attitude to the intervention was assessed with a short questionnaire covering practical issues of completing computer questionnaires in clinics, questionnaires' content, patient beliefs whether their functioning should be considered by the doctors and the overall usefulness of the intervention for their care (Appendix 1).

#### Clinicians

The clinical usefulness of the QL information was discussed with the physicians after each intervention consultation using semi-structured interviews based on a questionnaire used by Wagner *et al* (1997). The interviews covered the following issues: (1) quality of the information gathered with the standard questionnaires –whether the QL scores provided any new information, information confirming doctor's knowledge, information conflicting with the clinical assessment, accurate information and clinically relevant of QL information; (2) usefulness of information during the consultation – general question on usefulness and for which part of the consultation, usefulness for communication and usefulness for the management of the patient; (3) perceived prolongation of the intervention consultations and by how many minutes; and (4) preferences for format of presentation of QL data (numerical or graphic). The questions on the quality of QL information and clinical usefulness had a suggested 5-points response format – not at all, a little, somewhat, quite a bit and very much, but clinicians were encouraged to provide further comments.

An end-of-study meeting was conducted with the three doctors together to discuss their experiences during the study. The following topics were covered: opinion on QL information, clinical usefulness, integration of QL data into the consultations, length of consultations, presentation of QL results and training of clinicians. The discussion was taped and transcribed.

### Statistical analysis

Wilcoxon signed rank test was used to compare: (1) the number of baseline and intervention visits when each of the seven possible discussion topics were included; (2) the overall number of topics discussed during the baseline and the intervention consultations; and (3) patient satisfaction with the baseline and the intervention consultations. The data from doctors' interviews and patients' attitude to QL questionnaires were analyzed descriptively. The end-of-study group discussion with clinicians was subjected to qualitative thematic analysis. The transcript was carefully reviewed independently by two researchers (G Velikova and AB Smith) for key phrases. Phrases were organized in clusters, compared with each other and a set of core themes was derived (Miles and Huberman, 1994).

## RESULTS

### Patients' characteristics

Forty-two patients were asked to take part in the study. Eight patients (19%) refused to participate (two stated that they did not like answering questions, one was taking part in a drug trial involving completion of QL questionnaires and five did not give a reason for refusal). Two patients completed the baseline assessment but refused the 2nd assessment (one felt that the questions were not relevant and the other was too ill to continue) and four did not attend for another clinic appointment during the study period due to changes in treatment plans. The analysis is based on 28 patients completing both parts of the study, 22 females and six males with median age 57.4 years (range 43–77 years). Eighteen patients had ovarian cancer and 10 patients had malignant melanoma. They were receiving either chemotherapy (24 patients) or biological therapy (four patients). Twenty-two patients were married/cohabiting, four were divorced/widowed and two did not endorse this item. Thirteen patients had basic school education, nine studied in college, three had higher university education and three missed this question. Fourteen patients were retired, six continued to work full or part time, four were homemakers, two checked other (education) and two responses were missing. The age and gender of the refusing patients and of the patients who did not complete the study was not significantly different from those of the participating patients (data not shown).

All patients completed the computer questionnaires during the clinic waiting time, usually after they had routine blood samples taken and were waiting to see the doctor. For the intervention visit the print-out of the results was attached to the front of the medical notes. Summary statistics of the EORTC QLQ-C30 and HADS results are presented in Tables 1

**Table 1:**
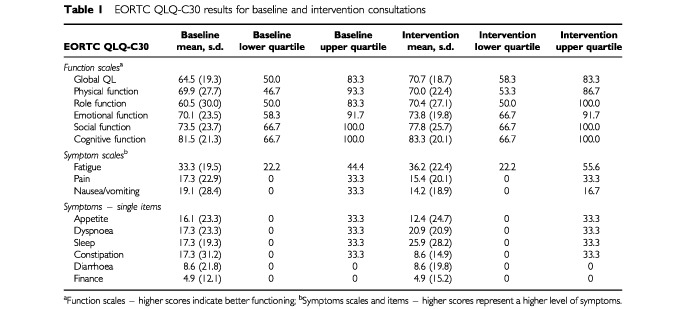
EORTC QLQ-C30 results for baseline and intervention consultations

and 2

**Table 2:**
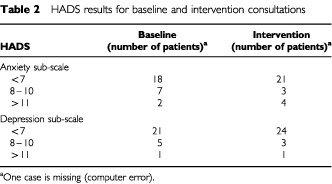
HADS results for baseline and intervention consultations

.

### Patient perceptions of the content of the consultations

Table 3

**Table 3:**
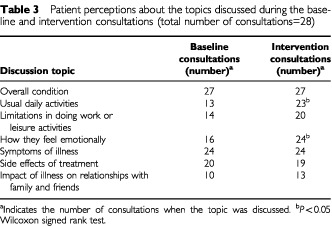
Patient perceptions about the topics discussed during the baseline and intervention consultations (total number of consultations=28)

presents patient opinion on what topics were discussed during their consultations. Patients felt that if clinicians had the QL results they enquired more often about usual daily activities (23 of the intervention *vs* 13 of the baseline consultations, Z=−2.71, *P*=0.007) and emotional problems (24 of the intervention *vs* 16 of the baseline consultations, Z=−2.11, *P*=0.035). Physicians also discussed more often limitations in doing work or leisure activities (20 intervention *vs* 14 baseline consultations), but the difference was of borderline significance (Z=−1.89, *P*=0.058).

**Figure 2:**
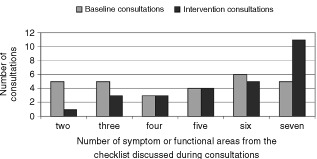
Number of areas from the checklist discussed during baseline and intervention consultations (based on 27 patients as one patient did not return the post intervention questionnaire and was excluded from this analysis).

There was a small increase in the overall number of issues discussed during the intervention consultation (Z=−1.89, *P*=0.059). Figure 2

shows the distribution of the number of topics covered in baseline and intervention consultations. During the intervention visit there was an increase of the consultations when all seven topics were included and a decrease of the encounters when only two topics were covered.

### Patient satisfaction with communication

We observed high patient satisfaction with both consultations and no difference between the two consultations (median and range for baseline and intervention consultations were 82.5, 74–85 and 83.5, 70–85 respectively). The range of possible total scores on the instrument is from 17 to 85. No difference was found either when comparing the three different dimensions of satisfaction with communication. The scores for baseline and intervention consultations on each of the three factors were respectively: Satisfaction with rapport (possible range 6–30) – median 30, range 26–30 and median 30, range 18–30; Dissatisfaction with doctor's manner (possible range 6–30) – median 30, range 28–30 and median 30, range 29–30; Understanding (possible range 4–20) – median 20, range 16–20 and median 20, range 17–20.

### Patient acceptance and attitude to QL intervention

Twenty-six patients completed the end-of-study questionnaires (Appendix 1). Two patients did not return the questionnaires. For presentation of results the response categories Definitely and Probably Yes/No were combined. All patients who returned the questionnaires were happy to do the computer questionnaires and their visit was not prolonged or made more difficult by the study. Only one patient felt that the standard QL questionnaires were not asking the right questions. The majority of the patients felt that the doctors considered their usual daily activities (*n*=23), how they feel emotionally (*n*=22) and their overall quality of life (*n*=26) when advising them. They wanted the doctors to ask them about their usual daily activities (*n*=20), feelings (*n*=23) and overall quality of life (*n*=24). Twenty-three patients felt the touch-screen questionnaires were useful to tell the doctor how they felt physically and emotionally and 24 were willing to complete them at each hospital visit as part of their usual care. Twenty-three patients thought this personal information should be kept in their medical notes, but only half (*n*=13) wanted to see the print-out of their questionnaire results.

### Clinical usefulness of the QL data from physician*'*s point of view

#### Interviews after each intervention consultation (*n*=28)

The clinicians discussed the QL results in 25 and did not discuss them in three consultations. On those three occasions the doctors either knew the patient well or had seen him/her very recently. The quantitative results from the semi-structured interviews with the physicians after each intervention consultation are presented in Table 4

**Table 4:**
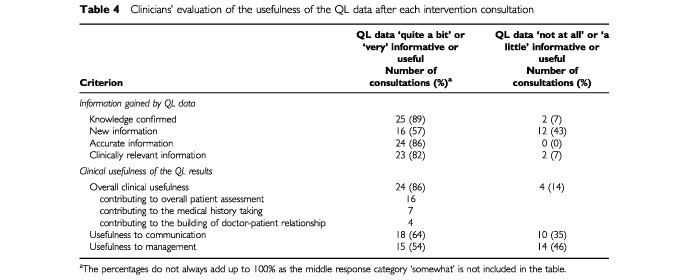
Clinicians' evaluation of the usefulness of the QL data after each intervention consultation

. Although the doctors knew the majority of the study patients well – 22 out of 28 (79%), the QL data still provided new information in half of the cases. The information was accurate and consistent with the medical assessment. There were four occasions when the responses to some questions were somewhat different from the clinical impression. In the first case the physician identified significant insomnia and shortness of breath which were not detected by the questionnaire. This patient needed a lot of help while completing the questionnaire and obviously had problems understanding some of the questions. The second case was a clinically anxious patient who had ‘normal’ scores. In this case the patient's spouse had dictated the responses. In the other two cases the QL results suggested symptoms (shortness of breath and pain) which were not considered to be serious by the physician.

The doctors felt that the QL data enhanced communication with the patients and contributed to some of the management decisions. The changes suggested by QL data consisted of stopping chemotherapy (*n*=1), readjusting symptomatic drugs (*n*=2), blood transfusion (*n*=1), counselling about life style (*n*=3), reassurance (*n*=1), discussion of depression (*n*=1).

The physicians felt that discussion of QL results may have lengthened nine of all 28 consultations. They estimated the prolongation to be between 1 and 5 min (median 3 min) and considered this acceptable.

#### End of study discussion

The qualitative content analysis of the discussion with the three participating clinicians identified a number of core themes. The doctors felt that the available information broadened the range of the inquiry and also helped them to focus their questions on relevant problems. Using the QL data in the encounters helped in building rapport with the patients and improved communication by giving patients chance to talk and by adding to the probing about less physical aspects. Clinicians were less clear how much the data influenced their decisions on patient management. All doctors commented that there would be times when the information may trigger a referral to other services (psychologists, social workers).

The clinicians felt that having symptoms and functional problems expressed quantitatively on a scale could be very useful especially for detection of change over time. There was a strong preference for the graphic format of presentation as it allowed to see changes at a single glance.

The physicians expressed concerns that the use of this rather broad, additional data may increase their workload.

## DISCUSSION

Our study confirms that computer-based individual QL assessment in oncology clinics with immediate feedback of results to clinicians is possible and feasible. The measurement procedure was integrated into the usual clinic routine and all patients completed the task within their waiting time.

We found that the QL data may have a positive effect on doctor-patient interactions by highlighting additional areas for discussion during the consultation. Even in these small numbers we showed a significant increase in the enquiries about daily activities and emotional functioning. It is important to emphasize that this is an increase from the patients' point of view as we asked the patients to report what issues were discussed with them. The design of the study allowed us to show that patients can feel a significant difference in the content of their consultations if their doctors had structured QL information. Taenzer *et al* (2000) published similar findings from a randomized study of 53 lung cancer patients. In structured interviews after consultations, the patients reported that more issues were addressed when the doctors had QL results. Using direct observation of oncology consultations Detmar and Aaronson (1998) found that the QL information stimulated physicians to initiate discussions on wider aspects of functioning, but there was no increase in the patients' rating of physicians' awareness of their problems. Several large studies in chronic diseases also suggested that feedback of health status data may facilitate communication between patients and clinicians and enhance patients' care (Kazis *et al*, 1990; Rubenstein *et al*, 1995; Wagner *et al*, 1997).

Our initial hypothesis was that if the QL results stimulate doctors to discuss a broader range of issues during the consultation, this may result in higher patients satisfaction with doctor–patient communication and overall satisfaction with the visit. Therefore we attempted to measure this possible effect by a satisfaction questionnaire. We were aware that patient satisfaction with care questionnaires tended to yield highly positive responses (Fitzpatrick, 1991; Hall and Dorman, 1988). Therefore, we carefully chose a questionnaire which was specifically modified and tested in cancer patients and which focused more narrowly on satisfaction with communication during the last clinic visit. Unfortunately even with this questionnaire, we observed that 25 out of 27 patients (one missing questionnaire) had very high satisfaction scores, above 79 (from a possible range 17–85), which represents a significant ‘ceiling’ effect. This makes the detection of improvement difficult. Taenzer *et al* (2000) reported very similar findings of high satisfaction in their group of lung cancer patients. Indeed, it may be that the concept of satisfaction is not an appropriate outcome measure in patients with advanced cancer with relatively short prognosis. People with serious diseases often give more favourable evaluation of medical care (Ben-Sira, 1980). Our patients attended a cancer centre with a concentration of experience and expertise in their problems. The patients understood that the options for treatment at this stage are limited and they were grateful for every help and support that was offered. We feel that high satisfaction with care among ill cancer patients is a psychological phenomenon that can not be avoided and should be taken into account when planning future research into patient care.

The computer measurement was well accepted and the patients who took part in the study felt that the questionnaires were a useful tool to tell the doctors about their feelings and were happy to complete them as part of their care. These positive results should be interpreted with caution as they reflect the opinion only of patients who completed the end of study questionnaire. We approached 42 patients and eight of them refused to take part. Two of those eight said that they did not like answering questions. One patient refused the 2nd assessment because the questions were not applicable to his situation. Two patients completed the 2nd intervention questionnaires in clinic, but did not fill in the end of study questionnaire asking for their opinion about the procedure. Therefore, we should assume that for those non-compliant patients the procedure might have been burdensome and not worthwhile. Overall, we feel that the proportion of patients who refused to participate (eight patients initially and two patients later i.e. 24%) and the proportion of patients completing both parts of the study (28 out of 42 approached i.e. 67%) is similar to therapeutic studies using QL measures (Osoba, 1994). However, our experience from this and other QL studies suggests that there will always be a number of patients (approximately 20% of all clinic attendants) for whom this approach of collecting QL information may not be acceptable or suitable. These are likely to be people who are physically very ill, emotionally distressed or simply not interested. In addition, the clinicians chose not to use the QL information in three out of 28 patients (10%), because they either knew them very well or had seen them recently.

We believe that one of the major reasons for not applying QL data in clinical practice is the lack of research data on its clinical usefulness in individual patients and the lack of guidelines how to integrate the data into the decision-making framework of a medical consultation (Till *et al*, 1994). Therefore, in this research project we placed an emphasis on detailed exploration of clinicians' assessment of the value of QL information, how they used the data during medical encounters and what were their needs for effective use of the data. The three doctors who took part in the study felt that the QL information was accurate, consistent with their medical assessment and clinically relevant. This practical observation is quite important and suggests that health surveys can be used reliably in clinical practice to gather information about individuals despite the methodological concern that they may not be sufficiently precise (McHorney and Tarlov, 1995).

The main contribution of the QL data was in bringing additional information and broadening the range of inquiry. The main impact on the consultation was in helping building rapport and better communication with the patients. The QL results were felt to be useful for exchanging information with other members of the team and for referrals to other services. The opportunity to have problems expressed on a scale and over time was rated as particularly valuable.

The doctors expressed two major concerns. They were apprehensive that the broad additional information may increase their workload. They also recognized how difficult it is to change their set pattern of questioning and behaviour during a medical consultation and include a new intervention. The doctors tended to follow the usual structure of a medical encounter and included the QL data towards the end to cover additional areas and give the patients the opportunity to talk.

One of the limitations of this study is the lack of direct observation of the consultations. We felt that any method of observation (i.e. tape or video recording or direct observation) may have an impact on the consultations and patients' and doctors' responses to the study questions. Therefore we decided to focus on patients' and doctors' perception and opinion about what happened during the medical encounters. However we recognize that an in-depth study of the effect of QL data on patient care would require formal analysis of the content of the consultations and we are using this approach in other studies.

Our results are encouraging but should be interpreted with caution at this stage as they are based on a small sample of patients and doctors. However they do suggest a potential for influencing doctor-patient interactions when using computer-administered standard QL questionnaires. It is unclear yet whether this broadening of the clinical enquiry will improve the process of care and whether it will bring benefits for the patients like better detection of morbidity, better control of symptoms or better emotional adjustment to cancer. Based on our results and on the findings of other researchers, we believe that this approach deserves further investigation and we are currently conducting a large randomized prospective clinical trial assessing the impact of QL information on the process and outcomes of medical care.

## Figures and Tables

**Figure 3:**
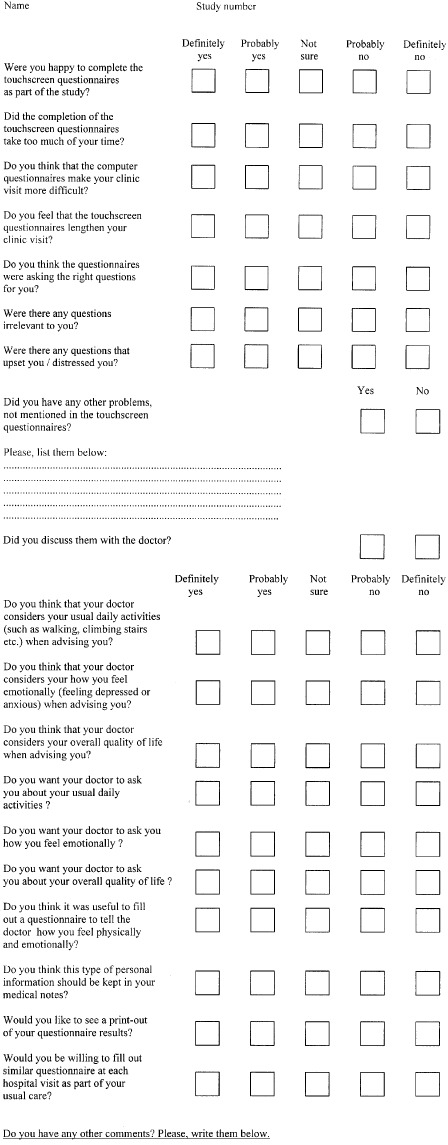
Questionnaire

## Appendix 1 - Quality of life measurement in daily oncology practicePatients’ Attitude Questionnaire

You may remember when we first discussed with you the quality of life study that it would be helpful to have feedback from those who participated. This information will give us clearer idea about how acceptable and useful routine collection of quality of life data is to patients.

Please answer all of the questions by ticking the appropriate box. If you have any other comments about this study please write them in the space provided and the end of the questionnaire. Thank you for your help. Figure 3

**Thank you very much for completing this questionnaire**

## References

[bib1] AaronsonNKAhmedzaiSBergmanBBullingerMCullADuezNJFilibertiAFlechtnerHFleishmanSBde HaesJC1993 The European Organization for Research and Treatment of Cancer QLQ-C30: a quality-of-life instrument for use in international clinical trials in oncology J Natl Cancer Inst 85 365376 8433390 10.1093/jnci/85.5.365

[bib2] Ben-SiraZ1980 Affective and instrumental components in the physician-patient relationship: an additional dimension of interaction theory J Health Soc Behav 21 170180 7391531

[bib3] CalkinsDRRubensteinLVClearyPDDaviesARJetteAMFinkAKosecoffJYoungRTBrookRHDelbancoTL1991 Failure of physicians to recognize functional disability in ambulatory patients Ann Intern Med 114 451454 1825267 10.7326/0003-4819-114-6-451

[bib4] CalkinsDRRubensteinLVClearyPDDaviesARJetteAMFinkAKosecoffJYoungRTBrookRHDelbancoTL1994 Functional disability screening of ambulatory patients: a randomized controlled trial in a hospital-based group practice J Gen Intern Med 9 590592 7823232 10.1007/BF02599291

[bib5] CohenJ1988 Statistical power analysis for behavioural sciences.Hillsdale NJ: Laurence Erlbaum Associates

[bib6] DetmarSBAaronsonNK1998 Quality of life assessment in daily clinical oncology practice: a feasibility study Eur J Cancer 34 11811186 9849476 10.1016/s0959-8049(98)00018-5

[bib7] DeyoRAPatrickDL1989 Barriers to the use of health status measures in clinical investigation, patient care, and policy research Med Care 27 S254S268 2646491 10.1097/00005650-198903001-00020

[bib8] FayersPMHopwoodPHarveyAGirlingDJMachinDStephensR1997 Quality of life assessment in clinical trials-guidelines and a checklist for protocol writers: the UK Medical Research Council experience Eur J Cancer 33 2028 9071894 10.1016/s0959-8049(96)00412-1

[bib9] FayersPWeedenSCurranDon behalf of EORTC Quality of life Study Group1998 EORTC QLQ-C30 Reference ValuesInternal publicationBrussels: EORTC

[bib10] FitzpatrickR1991 Surveys of patients satisfaction: I-Important general considerations BMJ 302 887889 1821624 10.1136/bmj.302.6781.887 PMC1669267

[bib11] FitzpatrickRFletcherAGoreSJonesDSpiegelhalterDCoxD1992 Quality of life measures in health care. I: Applications and issues in assessment BMJ 305 10741077 1467690 10.1136/bmj.305.6861.1074 PMC1883623

[bib12] HallJDormanMC1988 What patients like about their medical care and how often they are asked: a meta-analysis of the satisfaction literature Soc Sci Med 27 935939 3067368 10.1016/0277-9536(88)90284-5

[bib13] HjermstadMJFayersPMBjordalKKaasaS1998 Health-related quality of life in the general Norwegian population assessed by the European Organization for Research and Treatment of Cancer Core Quality-of-Life Questionnaire: the QLQ=C30 (+ 3) J Clin Oncol 16 11881196 9508207 10.1200/JCO.1998.16.3.1188

[bib14] KazisLECallahanLFMeenanRFPincusT1990 Health status reports in the care of patients with rheumatoid arthritis J Clin Epidemiol 43 12431253 2243259 10.1016/0895-4356(90)90025-k

[bib15] McHorneyCATarlovAR1995 Individual-patient monitoring in clinical practice: are available health status surveys adequate? Qual Life Res 4 293307 7550178 10.1007/BF01593882

[bib16] MilesMBHubermanAM1994 Early steps in analysisInQualitative data analysis: an expanded sourcebook,Miles MB, Huberman AM (eds)pp 5089Thousand Oaks, London, New Delhi: Sage Publications

[bib17] OsobaD1994 Lessons learned from measuring health-related quality of life in oncology J Clin Oncol 12 608616 8120561 10.1200/JCO.1994.12.3.608

[bib18] PassikSDDuganWMcDonaldMVRosenfeldBTheobaldDEEdgertonS1998 Oncologists' recognition of depression in their patients with cancer J Clin Oncol 16 15941600 9552071 10.1200/JCO.1998.16.4.1594

[bib19] RubensteinLVMcCoyJMCopeDWBarrettPAHirschSHMesserKSYoungRT1995 Improving patient quality of life with feedback to physicians about functional status J Gen Intern Med 10 607614 8583263 10.1007/BF02602744

[bib20] SigleJPorzsoltF1996 Practical aspects of quality-of-life measurement: design and feasibility study of the quality-of-life recorder and the standardized measurement of quality of life in an outpatient clinic Cancer Treat Rev 22Suppl A7589 10.1016/s0305-7372(96)90067-5 8625353

[bib21] TaenzerPASpecaMAtkinsonMJBultzBDPageSHarasymPDavisJL1997 Computerized quality-of-life screening in an oncology clinic Cancer Pract 5 168175 9171553

[bib22] TaenzerPBultzBDCarlsonLESpecaMDeGagneTOlsonKDollRRosbergerZ2000 Impact of computerized quality of life screening on physician behaviour and patient satisfaction in lung cancer outpatients Psychooncol 9 203213 10.1002/1099-1611(200005/06)9:3<203::aid-pon453>3.0.co;2-y 10871716

[bib23] TillJEOsobaDPaterJLYoungJR1994 Research on health-related quality of life: dissemination into practical applications Qual Life Res 3 279283 7812281 10.1007/BF00434902

[bib24] VelikovaGWrightEPSmithABCullAGouldAFormanDPerrenTSteadMBrownJSelbyPJ1999 Automated collection of quality of life data: a comparison of paper and computer-touchscreen questionnaires J Clin Oncol 17 9981007 10071295 10.1200/JCO.1999.17.3.998

[bib25] WagnerAKEhrenbergBLTranTABungayKMCynnDJRogersWH1997 Patient-based health status measurement in clinical practice: a study of its impact on epilepsy patients' care Qual Life Res 6 329341 9248315 10.1023/a:1018479209369

[bib26] WilkinDHallamLDoggettMA1992 Measures of need and outcome for primary health care.Oxford: Oxford University Press

[bib27] WareJrJE1995 The status of health assessment 1994 Ann Rev Publ Health 16 327354 10.1146/annurev.pu.16.050195.001551 7639876

[bib28] ZigmondASSnaithRP1983 The Hospital Anxiety and Depression Scale Acta Psychiatr Scand 67 361370 6880820 10.1111/j.1600-0447.1983.tb09716.x

